# Supplementary material for: Time‑resolved multi-omic analysis of paclitaxel exposure in human iPSC‑derived sensory neurons unveils mechanisms of chemotherapy‑induced peripheral neuropathy
Source: Cell Death Dis. 2026 Feb 10;17(1):211. doi: 10.1038/s41419-026-08445-2 (PMC12921266; doi:10.1038/s41419-026-08445-2)
Supplement: Supplementary file 15 — Dataset 1. [file 41419_2026_8445_MOESM15_ESM.zip › CDD_Open_Data/04_RNA_Seq/Timeline_panel_plots_etc_2023-01-23_1609.zip!/Panel_Plots_etc/Evidence_Plots/72h/evidence_plot_standard contrasts_msigdb_hallmark_72h_PTX_vs_DMSO_ID0_M5908_HALLMARK_ANDROGEN_RESPONSE.pdf]

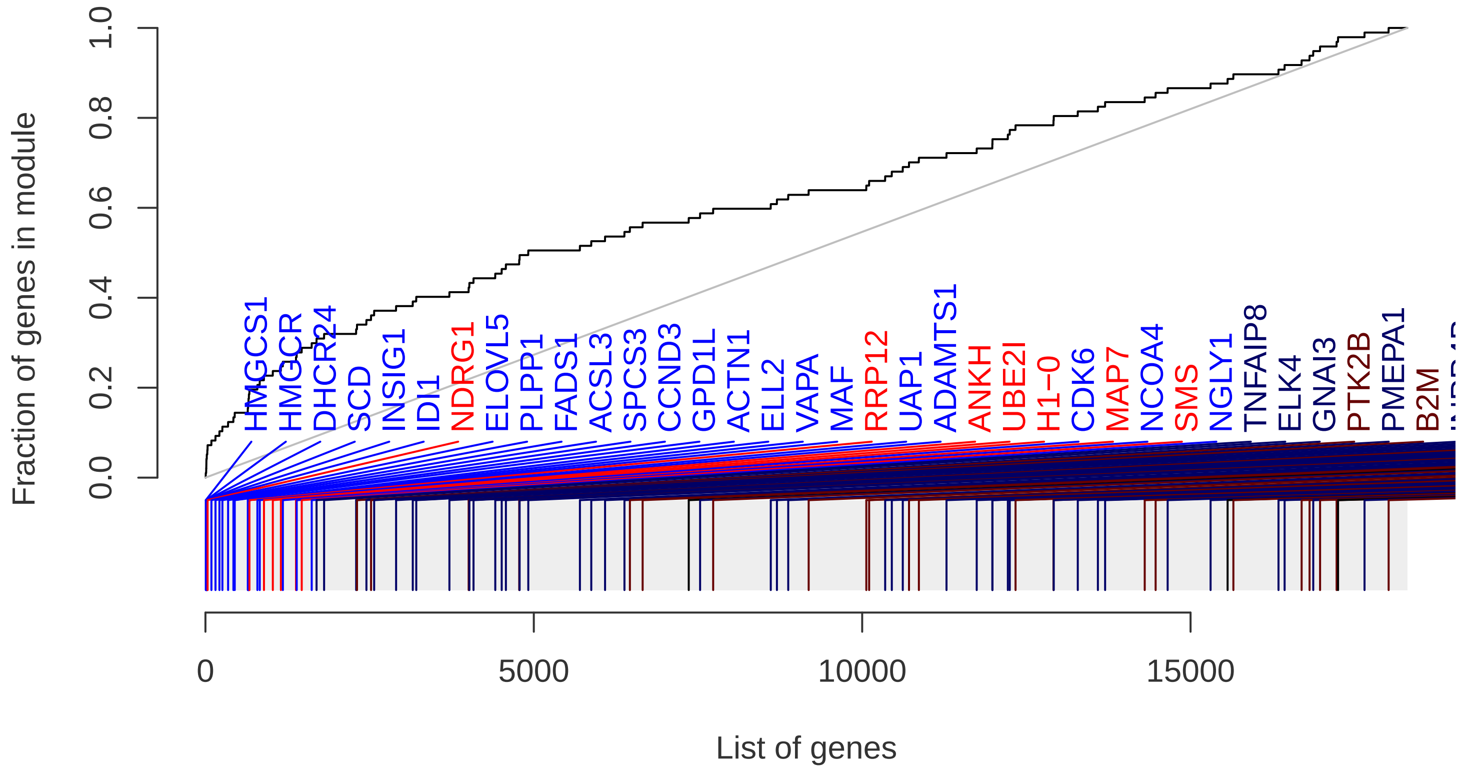

Fraction of genes in module
0.0
0.2
0.4
0.6
0.8
1.0
List of genes
0
5000
10000
15000
HMGCS1
HMGCR
DHCR24
SCD
INSIG1
IDI1
NDRG1
ELOVL5
PLPP1
FADS1
ACSL3
SPCS3
CCND3
GPD1L
ACTN1
ELL2
VAPA
MAF
RRP12
UAP1
ADAMTS1
ANKH
UBE2I
H1−0
CDK6
MAP7
NCOA4
SMS
NGLY1
TNFAIP8
ELK4
GNAI3
PTK2B
PMEPA1
B2M
